# Predictive factors of non-deterioration of glucose tolerance following a 2-year behavioral intervention

**DOI:** 10.1186/1758-5996-2-52

**Published:** 2010-07-30

**Authors:** Bianca Almeida-Pititto, Amélia T Hirai, Daniela S Sartorelli, Helena A Harima, Suely GA Gimeno, Sandra RG Ferreira

**Affiliations:** 1 Department of Nutrition, School of Public Health, University of Sao Paulo-Av. Dr. Arnaldo, 715, CEP 01246-904, São Paulo-SP, Brazil; 2 Department of Preventive Medicine, Federal University of Sao Paulo-Rua Borges Lagoa, 564, cj. 81/82, Vila Clementino CEP 04038-000, São Paulo, SP, Brazil; 3 Department of Social Medicine, University of Sao Paulo-Rua Onze de Agosto, 798 apto. 81, Campos Elíseos, CEP 14085-030, Ribeirão Preto, SP, Brazil

## Abstract

**Aim:**

To identify predictive factors associated with non-deterioration of glucose metabolism following a 2-year behavioral intervention in Japanese-Brazilians.

**Methods:**

295 adults (59.7% women) without diabetes completed 2-year intervention program. Characteristics of those who maintained/improved glucose tolerance status (non-progressors) were compared with those who worsened (progressors) after the intervention. In logistic regression analysis, the condition of non-progressor was used as dependent variable.

**Results:**

Baseline characteristics of non-progressors (71.7%) and progressors were similar, except for the former being younger and having higher frequency of disturbed glucose tolerance and lower C-reactive protein (CRP). In logistic regression, non-deterioration of glucose metabolism was associated with disturbed glucose tolerance - impaired fasting glucose or impaired glucose tolerance - (p < 0.001) and CRP levels ≤ 0.04 mg/dL (p = 0.01), adjusted for age and anthropometric variables. Changes in anthropometry and physical activity and achievement of weight and dietary goals after intervention were similar in subsets that worsened or not the glucose tolerance status.

**Conclusion:**

The whole sample presented a homogeneous behavior during the intervention. Lower CRP levels and diagnosis of glucose intolerance at baseline were predictors of non-deterioration of the glucose metabolism after a relatively simple intervention, independent of body adiposity.

## Introduction

Several anthropometric and metabolic parameters have been associated with cardiometabolic risk [1]. More recently, inflammatory markers have been studied as predictors of diabetes and cardiovascular events [2]. Japanese-Brazilians are considered as high cardiometabolic risk since they present high prevalence of disturbances of glucose metabolism and other cardiovascular risk factors [3,4]. Environmental factors should contribute to such picture and certain dietary habits were previously shown to predict metabolic syndrome in this population [5]. Therefore, a behavioral intervention was developed in order to improve cardiometabolic profile of this Japanese-Brazilian population.

Prospective randomized controlled studies have shown that behavioral interventions targeting diet and physical activity can delay or prevent the progression from impaired glucose tolerance (IGT) to diabetes [6-8]. However, the effect of lifestyle intervention varies among individuals and it would be helpful to identify those who benefit from an intervention and therefore should be targeted considering such strategy.

The Diabetes Prevention Study (DPS) showed the baseline characteristics of individuals who had benefits from the intervention [9]. However, intensive interventions as those employed in the DPS might not be feasible in developing countries. The Japanese-Brazilian Diabetes Study (JBDS) Group implemented an intervention based on lifestyle counseling for diabetes prevention, using resources tailored to the reality of public health in a developing country. Preliminary results showed that dietary n-3 fatty acid may predict improvement in glucose disturbance after the first year of intervention [10].

We investigated the participants who had benefits from the intervention, maintaining or improving their glucose tolerance status. The objective of this study was to identify predictive factors of those who benefit from the intervention regarding non-deterioration of glucose tolerance status following 2-year behavioral prevention program in a developing country. Additionally, we compared lifestyle habits and anthropometry after the intervention of those who improved or maintained glucose tolerance status with those who worsened it.

## Methods

### ■ Study design

First- (Japan-born) and second-generation (Brazil-born) Japanese-Brazilians, who participated in the Study on Diabetes and Associated Diseases in Japanese-Brazilian Population conducted in 2000, in the city of Bauru (IDH-M 0.825) [11]; State of Sao Paulo, Brazil, were invited to join a 2-year non-pharmacological intervention program, started in 2005. The aims and potential benefits of the behavioral intervention were outlined in invitation letters and telephone contacts. From a total of 728 who agreed to participate, 413 individuals completed the intervention. After excluding individuals who already had a diagnosis of diabetes or were taking hypoglycemic medication, 254 participants were included. Baseline demographic and clinical characteristics of the participants in 2005 who were lost to follow-up (n = 315) were similar to those who completed the 2-year intervention (data not shown).

The Institutional Ethics Committee approved the study protocol and written consent was obtained from all participants.

### ■ Intervention

The intervention strategy targeted changes in dietary intake and levels of physical activity [12], and was based on a previous trial conducted among overweight Brazilian adults [13]. Participants were scheduled for one individualized dietary counseling, five group sessions for nutritional and physical activity recommendation and two community events of exercise - including walking, stretching and dancing - during the two-year intervention program. In the individualized dietary counseling session, participants had a face-to-face one-hour consultation session with a nutritionist (60 days after the first assessment), during which they received a diet prescription with a food exchange list and were encouraged to practice at least 30 minutes of physical activity every day. Group sessions included 10 participants and were coordinated by nutritionists and physical educators. The relatives of all participants were also invited to take part in the group sessions. During the group sessions, physical educators and dietitians reinforced the role of a healthy lifestyle and discussed the barriers to reaching the goals of the intervention program.

The dietary recommendation consisted of changes in total energy intake according to the individual nutritional status. A low calorie diet was prescribed (restriction of 500 to 1000 calories daily from the estimated total energy expenditure) for overweight individuals or those with central obesity but not for normal weight individuals. The proportions of macronutrients in relation to total energy intake were 50-60% of carbohydrates; < 30% of total fat; < 10% of saturated fat; 10-15% of proteins; < 300 mg of cholesterol and ≥ 15 g of vegetable fibers.

The goals of the intervention were ≥ 5% weight loss for those with excessive weight, ≥ 150 minutes/week of physical activities, daily intake < 10% of saturated fat; daily consumption of ≥ 400 g of fruits and vegetables.

### ■ Clinical and laboratory procedures

Participants were scheduled for laboratory procedures and visits with the multi-professional team in the nutritional clinics of the Sagrado Coração University, in the city of Bauru at baseline and after one and two years of the intervention program. In each occasion, fasting blood samples (and also an oral glucose tolerance test with 75 g of glucose) were obtained for biochemical and hormonal determinations. Individuals underwent a detailed medical examination, the International Physical Activity Questionnaire was applied and 3-day food records were obtained.

Body weight and height were measured using calibrated electronic scales and a fixed rigid stadiometer, respectively, while participants wore light clothing without shoes. Body mass index (BMI) was calculated as weight (kilograms) divided by height (meters) squared. Waist circumference was measured with an inextensible tape, according to WHO technique [14]. The cutoffs to diagnose overweight (BMI ≥ 23 kg/m^2^) and central obesity (abdominal circumference ≥ 80 cm for women and ≥ 90 cm for men) were based on the WHO criteria for Asian populations [15].

Blood pressure was taken three times using an automatic device (Omron model HEM-712C, Omron Health Care, Inc, USA), after a five-min rest in sitting position. Blood pressure was considered as the mean of the second and third measurements. Hypertension was diagnosed if the systolic blood pressure was ≥ 140 mmHg and/or diastolic blood pressure ≥ 90 mmHg [16].

Fasting blood samples were taken and a 75 g oral glucose tolerance test performed. Samples were immediately centrifuged and analyses made in the local laboratory. Plasma glucose was measured by the gluco-oxidase method and lipoproteins enzymatically by automatic analyser. ADA criteria were used to diagnose diabetes, impaired fasting glucose (IFG) and impaired glucose tolerance (IGT) [17]; the latter two categories were grouped as glucose intolerance. The diagnosis of dyslipidemia was based on the National Cholesterol Education Program [18].

Remaining samples were stored at -80°C prior to hormone and inflammatory markers assay. Insulin was determined by immunometric assay using a quantitative chemiluminescent kit (Euro DPC Limited - Glyn Rhonwy, Llanberis, Caernarfon, Gwynedd, UK), with analytical sensitivity of 2.0 uIU/mL; intra-assay coefficient of variability ranged from 5.3% to 6.4% and the inter-assay coefficient of variability from 5.9% to 8.0%. Homeostasis model assessment of insulin resistance (HOMA-IR) was calculated [19]. Adiponectin was measured by radioimmunoassay (Linco Research Inc, St Charles, MO, USA). The high sensitivity C-reactive protein (CRP) was assessed by an immunometric assay with a quantitative chemiluminescent kit (DPC - Diagnostic Products Corporation- Los Angeles, USA) with an analytical sensitivity of 0.01 mg/dL and a functional sensitivity of less than 0.02 mg/dL; the intra-assay coefficient of variability (CV) ranged from 4.2% to 6.4% and the inter-assay CV from 4.8% to 10.0%. Interleukin 6 (IL-6) and tumor necrosis factor-alpha (TNF-α) were determined by immunometric assay a quantitative chemiluminescent kits (Euro DPC Limited - Glyn Rhonwy, Llanberis, Caernarfon, Gwynedd, UK). Analytical sensitivity of IL-6 was 2.0 pg/mL; its intra-assay CV ranged from 3.5% to 6.2% and the inter-assay CV from 5.1% to 7.2%. TNF-α analytical sensitivity was 1.7 pg/mL and intra-assay CV ranged from 2.6% to 3.6% and the inter-assay CVs from 4.0% to 6.0%.

### ■ Statistical analysis

Individuals who persisted in their categories of glucose tolerance or improved (changed from glucose intolerance to normal tolerance) glucose metabolism from 2005 to 2007 were classified as "non-progressors", and those who worsened the glucose tolerance status (changed from normal to glucose intolerance or diabetes and from glucose intolerance to diabetes) as "progressors".

Baseline characteristics of the progressors and non-progressors are presented as qualitative variables (dichotomized for established cutoffs available or in tertiles) in table 1. Chi-square test was used for comparisons between these groups. Variables that showed statistically differences at the level of p < 0.20 were entered into a logistic regression model, in which non-progressor category was the outcome variable. Logistic regression analysis was performed to identify independent predictors of those who benefit from the intervention in terms of glucose tolerance status. The final model was adjusted for body adiposity, since increased BMI and abdominal circumference are relevant risk factors for deterioration of glucose metabolism.

**Table 1:** Baseline characteristics of participants according to the progression of glucose metabolism disturbance.

	Progressors n = 72	Non-progressors n = 182	p
Women	44 (61)	104 (57)	0.56
Age ≥ 60 years	42 (58)	84 (46)	0.08
Increased BMI	36 (50)	104 (57.1)	0.28
Abdominal obesity	45 (62.5)	104 (57.1)	0.46
Hypertension	31 (43)	77 (43.1)	0.91
Glucose metabolism			
Normal	47 (65.3)	41 (22.5)	<0.001
IFG or IGT	25 (34.7)	141 (77.5)	
Dyslipidemia	55 (76.4)	151 (83.0)	0.42
Insulin (uIU/mL)			
< 2.3	23 (41.1)	51 (30.0)	0.22
≥ 2.3 to < 4.8	23 (41.1)	73 (42.9)	
≥ 4.8	10 (17.9)	46 (27.1)	
Adiponectin (ng/mL)			
< 5.3	20 (33.2)	58 (33.9)	0.40
≥ 5.3 to < 8.8	17 (28.3)	62 (36.3)	
≥ 8.8	23 (38.3)	51 (29.8)	
C-reactive protein (mg/dL)			
< 0.04	11 (18.6)	49 (28.7)	0.02
≥ 0.04 to < 0.14	18 (30.5)	70 (40.9)	
≥ 0.14	30 (50.8)	52 (30.4)	
Interleukin-6 (pg/dL)			
< 1.8	14 (25.0)	51 (30.2)	0.76
≥ 1.8 to < 2.8	21 (37.5)	58 (34.3)	
≥ 2.8	21 (37.5)	60 (35.5)	
Tumor necrosis factor-α (pg/dL)			
< 3.6	18 (32.1)	53 (31.4)	0.98
≥ 3.6 to < 6.2	20 (35.7)	59 (34.9)	
≥ 6.2	18 (32.1)	57 (33.7)	

Data expressed in number of individuals and percentages in parenthesis.

p value from chi-square test.

BMI, body mass index

IFG, impaired fasting glucose

IGT, impaired glucose tolerance

To evaluate if those who benefit from the intervention in terms of glucose tolerance status had healthier dietary habits, physical activity or anthropometry after the intervention, we compared the percentages (by chi-square test) of individuals who succeeded in achieving each of the four study goals and the mean changes (Student *t *test) in abdominal circumference from 2005 to 2007. Considering the small number of participants achieving the goal of physical activity (5% of the whole sample), we compared the percentage of those who increased their physical activity at any degree after the intervention (43% of the whole sample).

Sensitivity analyses were conducted excluding those who were taking and/or changed medications (antihypertensive, lipid lowering or antidiabetic agents) which might influence outcomes.

Statistical analyses were performed using SPSS 12.0 software (SPSS Inc. Woking, Surrey, UK). Significance level was set at p < 0.05.

## Results

Of 254 non-diabetic participants [mean 59.4 (11.9) years], 59.7% were women and 83% second-generation Japanese-Brazilians. Considering their mean values (and standard deviation) of BMI [24.2 (4.0) kg/m^2^] and abdominal circumference [83.9 (9.9) cm for women and 90.1 (10.2) cm for men], they had increased body adiposity. After the intervention, there was a slight but significant mean (95% confidence interval) reduction in BMI [-0.3 (-0.2 to -0.4) kg/m^2^] and in abdominal circumference [-1.9 (-2.6 to 5.6) cm] in the whole sample.

The most common goal achieved was concerned to the saturated fat intake; 74.5% consumed less than 10% of saturated fat of total energy consumed per day. Almost 42% of the individuals reached ≥ 5% weight loss from the baseline; 39% achieved the goal of fruits and vegetables intake (> 400 g of per day) and only 7.4% reported to practice at least 150 minutes of physical activity per week. Stratifying in progressors and non-progressors, these proportions did not differ between subsets.

One hundred and three participants had the diagnosis of IGT or IFG in 2005. The intervention induced higher mean changes (and standard deviation) in fasting plasma glucose in individuals with IFG or IGT compared with those with normal glucose tolerance [- 3.2 (12.3) *vs*. 0.6 (11.6) mg/dL, respectively, p = 0.02].

A total of 72% (n = 182) of the participants maintained or improved their glucose tolerance status (non-progressors) following the intervention. Baseline characteristics of the non-progressors and progressors were similar, except for the higher percentage of individuals in the age group < 60 years, with glucose intolerance (IFG or IGT) category and in the lower tertiles of CRP levels (table 1). Looking at the mean values (and standard deviation) of these variables, the non-progressors had younger age [58.0 (11.7) *vs*. 61.2 (12.4) years, p = 0.05], higher fasting plasma glucose [100.7 (11.0) *vs*. 96.3 (10.5) mg/dL, p = 0.004] and lower CRP levels [0.20 (0.43) *vs*. 0.27 (0.38) mg/dL, p = 0.009] compared with the progressors (data not shown in table).

In the final logistic regression model, the diagnosis of glucose intolerance (IFG or IGT) and CRP levels < 0.04 mg/dL at baseline were predictive of non-deterioration of glucose tolerance following the intervention program, adjusted for age ≥ 60 years (table 2). The significance of these results did not change when elevated BMI or abdominal circumference or gender was entered in the model.

**Table 2:** Estimated odds ratios of non-progression (95% confidence intervals) in Japanese-Brazilians who underwent the intervention, obtained from logistic regression analysis.

	Odds ratio (95% CI)	P value
Age ≥ 60 years	0.50 (0.25-1.21)	0.06
Glucose intolerance*	5.89 (2.85-12.16)	<0.001
C-reactive protein		
≥ 0.14	1.00	
≥ 0.04 to < 0.14	2.1 (0.98-4.60)	0.06
< 0.04	3.3 (1.27-8.66)	0.01

* Includes impaired fasting glucose and impaired glucose tolerance

When abdominal circumference or BMI were entered in the model, the significant results did not change.

Sensitivity analyses, regarding the use and/or change in medications, did not change the results.

## Discussion

Reducing the high risk of cardiovascular disease among the Japanese-Brazilian population was the motivation for this behavioral intervention, which could potentially be extended into a broader prevention program throughout the country. In this relatively simple intervention program, most of the participants (71.7%) maintained or improved their glucose tolerance status after the intervention. This study was able to identify baseline characteristics associated with the prevention of the progression of the natural history of glucose metabolism disturbances in two years.

Descriptive analysis identified three baseline characteristics - age ≤ 60 years, presence of glucose intolerance and CRP levels ≤ 0.04 mg/dL - which differed between progressors and non-progressors. Those who improved or maintained glucose tolerance status were younger than those who deteriorated, but age was not independently associated with non-progressor status in logistic regression. This finding might indicate that age is an important factor for deterioration of glucose metabolism, but other metabolic characteristics may minimize the expected effect of aging. According to the multivariate analyses, a lower grade of inflammation (reflected by the CRP levels) could identify those more prone to benefit from a behavioral intervention in terms of non-deterioration of glucose tolerance. Also, individuals with glucose intolerance (IFG or IGT) had a better response to intervention in terms of glucose tolerance outcome. Such finding is in agreement with the DPS, in which individuals with more unfavorable baseline diabetes risk score (FINDRISC) had a higher decrease in diabetes incidence [9]. This may suggest that the impact of behavioral interventions on cardiometabolic profile is more pronounced in those with any degree of metabolic disturbances. In fact, a higher decrease in plasma glucose in these individuals was found when compared to normal glucose tolerant ones. We speculate that those with abnormalities of glucose metabolism may be more motivated to modify their lifestyle in order to improve their health, when submitted to prevention programs.

This finding of lower CRP level at baseline in the non-progressor group is in the same line of the results from prospective case-control studies, in which elevated levels predicted the development of type 2 diabetes, supporting a possible role for inflammation in diabetogenesis [20-23]. In our study, multivariate analysis showed that CRP levels were inversely and independently associated with the non-progression status, even adjusted for age, glucose tolerance status and measurements of adiposity. Among Japanese Americans, Nakanishi *et al *also found that CRP was a risk factor for development of type 2 diabetes, independently of either obesity or insulin resistance [2]. We suggest that the lower levels of CRP might be indicative of a better prognosis in terms of metabolic risk.

The goals of our community-based intervention program were not achieved by the majority of the participants. This could be attributed to our approach - tailored to the reality of a developing country - that was much less intensive than that used in other intervention trials [6,7]. Similar proportions of progressors and non-progressors achieved the goals. It means that these goals should be reflecting mainly the effect of the follow-up and reinforces the importance of identifying predictors at baseline. Interestingly, the most commonly achieved goal was the reduction of saturated fat intake. Considering previous findings in the same population, regarding the association of fat intake with metabolic syndrome [5], such reduction was highly desirable and could have contribute to the improvement in metabolic profile.

Slight, but significant reduction in anthropometry occurred following the intervention in the whole sample. The lack of association of the intervention-induced adiposity reduction on glucose metabolism benefits is in agreement with other studies conducted in Asian populations. The Indian Diabetes Prevention Program showed reduced risk of diabetes without any significant change in anthropometric variables [24]. Another study based on lifestyle intervention showed a greater reduction in the incidence of diabetes in Japanese men than might be expected simply on the basis of the decrease in BMI [25], suggesting that while weight loss may be desirable, it does not fully explain the effects of behavioral interventions.

Our study has limitations. The definition of progressors implicates in certain heterogeneity of this group of individuals, including not only 32 incident cases of diabetes, but also those who progressed from normal glucose tolerance to IFG or IGT. Another point to be observed is that we do not have the data of those individuals who were lost to follow-up that could implicate in a selection bias. However, baseline characteristics of the subset that did not complete the intervention program did not differ from those who were evaluated at the end of the intervention. The findings of the present study cannot be extrapolated to other populations.

In summary, our findings suggest that individuals with lower CRP levels and with glucose intolerance at baseline were the ones who will benefit from this relatively simple intervention in terms of glucose metabolism, independent of body adiposity. The homogeneous behavior of the whole sample during the intervention period suggests that the simple fact of being followed-up may result in favorable changes in glucose tolerance, which seems relevant in terms of public health for developing countries.

## Competing interests

The authors declare that they have no competing interests.

## Authors' contributions

BA participated in the intervention planning and execution and performed the statistical analysis. ATH participated in the design of the study and in the intervention execution. DSS participated in the design of the study and in the intervention execution. HAH participated in the design of the study and in the intervention execution. SGAG participated in the design of the study and in the intervention execution. SRGF participated in the design of the study and coordination and in the intervention execution.

All authors have read and approved the final manuscript.
